# Cyanobacteria blooms and non-alcoholic liver disease: evidence from a county level ecological study in the United States

**DOI:** 10.1186/s12940-015-0026-7

**Published:** 2015-05-07

**Authors:** Feng Zhang, Jiyoung Lee, Song Liang, CK Shum

**Affiliations:** 1 Environmental Science Graduate Program, The Ohio State University, Columbus, OH USA; 2 College of Public Health, Division of Environmental Health Sciences, The Ohio State University, Columbus, OH USA; 3 Department of Food Science and Technology, The Ohio State University, Columbus, OH USA; 4 Department of Environmental and Global Health, College of Public Health and Health Professions, University of Florida, Gainesville, FL 32610 USA; 5 Emerging Pathogens Institute, University of Florida, Gainesville, FL 32610 USA; 6 Division of Geodetic Science, School of Earth Sciences, The Ohio State University, Columbus, OH USA; 7 Institute of Geodesy & Geophysics, Chinese Academy of Sciences, Wuhan, China

## Abstract

**Background:**

Harmful cyanobacterial blooms present a global threat to human health. There is evidence suggesting that cyanobacterial toxins can cause liver damage and cancer. However, because there is little epidemiologic research on the effects of these toxins in humans, the excess risk of liver disease remains uncertain. The purpose of this study is to estimate the spatial distribution of cyanobacterial blooms in the United States and to conduct a Bayesian statistical analysis to test the hypothesis that contamination from cyanobacterial blooms is a potential risk factor for non-alcoholic liver disease.

**Methods:**

An ecological study design was employed, in which county-specific gender and age standardized mortality rates (SMR) of non-alcoholic liver disease in the United States were computed between 1999 and 2010. Bloom coverage maps were produced based on estimated phycocyanin levels from MERIS (Medium Resolution Imaging Spectrometer) water color imageries from 08/01/2005 to 09/30/2005. A scan statistical tool was used to identify significant clusters of death from non-alcoholic liver disease. A map of local indicator of spatial association (LISA) clusters and a Bayesian spatial regression model were used to analyze the relationship between cyanobacterial bloom coverage and death from non-alcoholic liver disease.

**Results:**

Cyanobacterial blooms were found to be widely spread in the United States, including coastal areas; 62% of the counties (1949 out of 3109) showed signs of cyanobacterial blooms measured with MERIS. Significant clusters of deaths attributable to non-alcoholic liver disease were identified in the coastal areas impacted by cyanobacterial blooms. Bayesian regression analysis showed that bloom coverage was significantly related to the risk of non-alcoholic liver disease death. The risk from non-alcoholic liver disease increased by 0.3% (95% CI, 0.1% to 0.5%) with each 1% increase in bloom coverage in the affected county after adjusting for age, gender, educational level, and race.

**Conclusions:**

At the population level, there is a statistically significant association between cyanobacterial blooms and non-alcoholic liver disease in the contiguous United States. Remote sensing-based water monitoring provides a useful tool for assessing health hazards, but additional studies are needed to establish a specific association between cyanobacterial blooms and liver disease.

## Background

Liver disease constitutes a rapidly increasing global burden to society and is an important cause of morbidity and mortality in the United States, accounting for up to 2% of all deaths in the US [1]. Economically, approximately 1% of the total national health care expenditure is spent on the care of patients with liver disease, which appears to be on the rise in the US [1]. In the United Kingdom, liver disease is the fifth most common cause of death with increasing mortality rates [2]. Major risk factors include hepatitis C and B viruses, heavy alcohol consumption, and non-alcoholic liver disease [3]. Non-alcoholic liver disease refers to a collection of liver diseases in people who drink little or no alcohol. Non-alcoholic liver disease is developing into a worldwide major health problem [2] with age, gender, and obesity as potential risk factors [4]. Accumulating evidence suggests that non-alcoholic liver disease is rapidly becoming another important cause of hepatocellular carcinoma [5].

Cyanobacterial blooms have been reported to be a severe problem in many water bodies and coastal areas around the world. Recent research suggests that eutrophication, coupled with climate change, promotes the worldwide proliferation and expansion of cyanobacterial harmful algal blooms [6]. These blooms can affect water quality, producing a variety of toxins, such as microcystins, nodularin, and anatoxin [7]. It has also been shown that the neurotoxic amino acid, beta-methylamino alanine (BMAA), is widely produced by cyanobacteria [8]. Human exposure to these toxins occurs through ingestion, skin contact, and inhalation [9]. Despite the potential health risks of cyanobacterial toxins, shown by animal studies, a limited number of epidemiological studies have been reported in humans. Microcystins usually accumulate in vertebrate liver cells and are suggested to cause liver damage [10], are the most common and more thoroughly studied of the cyanobacterial toxins, and have been identified as being hepatotoxins [11]. Microcystins are resistant to digestion in the gastrointestinal tract and are concentrated in the liver by an active transport system [12]. Acute poisoning results in destruction of the liver architecture, leading to blood loss in the liver and hemorrhagic shock [13]. Chronic exposure to these toxins causes an ongoing active liver injury in mice [14] and there is experimental evidence suggesting that microcystins can cause tumor promotion [15]. Human exposure to microcystins occurs through: 1) ingestion of microcystins from tap water due to cyanonbacterial blooms in the source water; 2) recreational exposure through accidental ingestion or inhalation and dermal contact; and 3) consumption of seafood with accumulated microcystin. Algal cells and waterborne toxins can be aerosolized by a bubble-bursting process via wind-driven, white-capped waves [16]. Aerosol samples, taken during recreational activities on bloom impacted lakes, have been found with detectable levels of microcystins [17,18]. Although the levels of aerosolized toxin were generally low, laboratory investigations have found that treatment of mice by the intranasal route to microcystin-LR, the most toxic known variant of microcystin, was an effective method for toxin exposure [19]. In addition, there is evidence that liver disease has been associated with the consumption of seafood, such as fish, and water contaminated with microcystin [20]. The relationship between cyanobacterial toxin and liver cancer has been presumed from several epidemiological studies in developing countries [21,22]. Deaths in Brazil have been attributed to exposure to cyanobacterial hepatotoxins (microcystins) via hemodialysis water [11] and chronic exposure to microcystins has been identified as a risk factor for childhood liver damage in China [23]. However, these and other epidemiological studies do not conclusively prove the etiological effect of cyantoxins, therefore, more studies are warranted to fully understand the health impact of these toxins.

In recent decades, the incidence and intensity of toxic cyanobacterial blooms, as well as the associated economic impact, have increased in the United States and worldwide [6,24]. Even though cyanobacterial blooms have become a serious problem for water resources in the United States, no federal regulatory guidelines for cyanobacteria or their toxins in drinking or recreational waters exist at this time. Some states such as Iowa, Minnesota, Nebraska, Wisconsin, California, Oregon and Ohio have established monitoring programs and routinely issue alerts for harmful cyanobacterial blooms [25]. Many states and other jurisdictions rely on WHO guidelines to manage cyanobacterial blooms and toxins, whereas other states have developted their own guidelines to support public health decision-making, such as posting advisories or closing access to contaminated water bodies [26]. Additional legislation is recommended to promote research and establish guidelines regarding cyanobacterial blooms [24].

Optical/Infrared remote sensing has been used to monitor algal blooms, mostly by quantifying the concentration of pigments in water bodies, such as chlorophyll-*a* (chl-*a*) or phycocyanin [27]. However, as chl-*a* is common to almost all phytoplankton, its retrieval from remotely sensed data cannot be used to specifically determine the abundance of cyanobacteria, especially where other groups of eukaryotic algae co-occur. In contrast to chl-*a*, phycocyanin is a pigment only found at high concentrations in cyanobacterial blooms; therefore phycocyanin has been shown to be a better indicator of cyanobacterial blooms [28] and has been proposed as a tool for inferring elevated microcystin levels [29]. Phycocyanin, other than chl-*a* and carotenoid, is the most measureable pigment-protein complex in *Microcystis* spp [30]. The Medium Resolution Imaging Spectrometer (MERIS) onboard the European Space Agency ENVIronmental SATellite (ENVISAT) is suitable for retrieving data on phycocyanin concentrations because one of its 15 VIS_NIR (Visible-Near Infrared) programmable spectral bands between 390 nm and 1040 nm, can be used to detect the phycocyanin absorption peak that is near 620 nm. ENVISAT MERIS on a push-broom detector generates an observation swath width of 1,150 km, and with an exact repeat-orbit of 35-days; the effective temporal sampling or Earth revisit time is 2–3 days with a spatial resolution of ~300 m. Different types of models, such as semi-empirical and single reflectance ratio, have been developed to quantify phycocyanin levels [31,32]. A nested semi-empirical band ratio model [33], based on MERIS, has been proven to statistically outperform other MERIS-based algorithms [30]. A previous study, based on Lake Erie beaches, also suggested that the semi-empirical band ratio model performed well even with relatively low phycocyanin levels [34]. Although MERIS ceased its operation on 9 May 2012, due to a sudden failure in communication in the ENVISAT satellite, it is still useful in terms of retrieving historical bloom conditions over Lake Erie back to 2002 when it was launched. The advantages of using satellite images for water quality parameters include: a) near continuous spatial coverage of satellite imagery allowing for estimates over large areas, and b) a record of archived imagery giving an estimation of historical bloom conditions. In other studies, the linkage between some satellite measured environmental factors and health risks showed a potential for satellite imagery use [35]. However, satellite imagery data have a limitation for near lake coastal regions because of land contamination, and the spatial (pixel) resolution of about 300 m.

The current study adopted an ecological method using aggregate disease mortality data at the county level for the contiguous US and MEIRS-derived data for cyanobacterial bloom coverage. Exploratory spatial analysis methods and regression models were used to test the hypothesis that non-alcoholic liver disease mortality rates are related to satellite-observed algal bloom coverage. The identification of cyanobacterial blooms as potential risk factors for non-alcoholic liver disease will help to address the prevention of this disease worldwide, including the US, and assist in drawing attention to mitigating cyanobacterial blooms throughout the world.

## Methods

First, coverage maps of cyanobacterial blooms in the US were produced using estimated phycocyanin levels from MERIS images. Second, standardized county level non-alcoholic liver disease mortality rates were computed using the mortality data from Multiple Cause of Death data. Subsequently, exploratory methods including spatial clustering and local indicator of spatial association were used to identify the linkage between cyaonobacterial blooms and non-alcoholic liver disease. A Bayesian regressional analysis was then used to quantitatively measure the linkage between cyanobacterial bloom and non-alcoholic liver disease mortality.

### MERIS-observed bloom coverage data

All MERIS L1B full resolution images covering the contiguous United States from 08/01/2005 to 9/30/2005 were retrieved from the National Aeronautics and Space Administration (NASA)’s Goddard Space Flight Center (GSFC) Ocean Color Science Team that are available from http://oceancolor.gsfc.nasa.gov/. August and September were chosen to match the common seasonal peak (late summer or early fall) of cyanobacterial blooms (http://www.cdc.gov/nceh/hsb/hab/default.htm). After downloading the data, we used the Basic ERS & Envisat (A) ATSR and MERIS (BEAM) VISAT toolbox provided by ESA and Brockmann Consult and its supplementary Regional Case-2 Water Processor [36] to further refine the data from the US. In particular, we applied the Case-2 Regional Processor (C2R) v1.5.2 to convert the top of atmosphere (TOA) radiance (archived in the original L1B data) to water leaving radiance (R_L_W_) above the surface. Phycocyanin levels were estimated using the nested semi-empirical band ratio model [33], which has proven to be quite reliable [37]. The nested semi-empirical band ratio model used MERIS bands 6, 7, 9, and 12. The absorption of phycocyanin, was calculated as below:

$$ {a}_{pc}(620)=\left\{\left[\left(\frac{B(705)}{B(620)}\right)\times \left({a}_w(709)+{b}_b\right)\right]-{b}_b-{a}_w(620)\right\}\times {\delta}^{-1}-\left(\varepsilon \times {a}_{chl}(665)\right) $$

In this model, the absobtion of chlorophyll *a* is calculated as:

$$ {a}_{chl}(665)=\left\{\left[\left(\frac{B(705)}{B(665)}\right)\times \left({a}_w(709)+{b}_b\right)\right]-{b}_b-{a}_w(665)\right\}\times {\gamma}^{-1} $$

Where B(X) = water-leaving reflectance centered at X nm [unit: dimensionless];

*a*_*w*_(665) = pure water absorption at 665 nm;

γ = 0.68, estimated chlorophyll*-a* absorption;

*a*_*w*_(709) = pure water absorption at 709 nm [unit: m^−1^];

*b*_*b*_ = backscattering coefficient estimated by a single band (Band 12 of MERIS in this study);

*a*_*w*_(620) = pure water absorption at 620 nm [unit: m^−1^];

δ = 0.82, correction factor; and

*ɛ*= 0.24

Maximum value composite technique was used to combine all images into one large image with each pixel being the highest value for that pixel location. This generates a cloud-free phycocyanin image for the spatial monitoring of cyanobacterial blooms over water bodies in the contiguous US.

Cyanobacteria blooms can present serious risks to human and animal health due to their ability to produce toxins. The World Health Organization (WHO) has provided guideline levels of 1 and 20 μg/L of microcystin in drinking and recreational water, respectively, in order to protect public health [38]. The WHO guideline for recreational exposure to cyanobacteria uses a three-tier approach based on cyanobacterial density and chl-*a* level [39]. For protection of health, due to the irritative or allergenic effects of cyanobacterial toxins, a guideline level of 20,000 cyanobacterial cells/ml (corresponding to 10 mg chl-*a*/liter under conditions of cyanobacterial dominance) has been derived [39]. We chose the level of 20,000 cyanobacterial cells/ml to be the threshold of significant blooms. To transform this threshold to a phycocyanin level, we used a linear relationship between the log transformed parameters suggested in Ahn et al. [40] and derived a level of 4.11 μg/L as equivalent to 20,000 cells/ml of cyanobacteria. The relationship between cyanobacterial cell abundance and phycocyanin is given as:

$$ \mathrm{Log}\left(\mathrm{cyanobacteria}\right)\kern0.5em =\kern0.5em 0.360\ast \log \left(\mathrm{phycocyann}\right)\kern0.5em +\kern0.5em 4.08 $$

As a precaution, 4 μg/L phycocyanin was used as our actual threshold for identifying water bodies affected by cyanobacterial blooms that could have potential adverse health effects on their neighborhoods. By overlaying the county boundary polygon GIS layer from US Census (http://www.census.gov/geo/maps-data/data/tiger-cart-boundary.html), with the MERIS bloom coverage imagery with a spatial resolution of 260 m × 290 m, (http://badc.nerc.ac.uk/data/meris/index-old.html), we calculated the bloom coverage as the percentage of county area covered by cyanobacterial blooms. The maximum value composite and the zonal statistics were performed using ArcGIS 10.0. Although the bloom coverage data are only from 2005, they were intended to represent the bloom distributions in that decade since the development of eutrophication and algal blooms is gradual over years [41,42] and studies show that eutrophication conditions in US estuaries remain nearly the same over a decade [43]. However, in some areas the cyanobacterial bloom situation may change rapidly due to eutrophication, mitigation or climate factors. Using only 2005 data may underestimate the bloom areas for the time period of 1999 to 2010 if the bloom situation increased exponentially during this time period.

### Non-alcoholic liver disease data

Non-alcoholic liver disease data (ICD-10 codes: R74.0, K71.0 – K77.8) [44] at the county level were extracted for the period from 1999–2010 from the Multiple Cause of Death data contained in the Centers for Disease Control and Prevention (CDC) Wide-ranging Online Data for Epidemiologic Research (WONDER) online database (http://wonder.cdc.gov/mcd.html). Non-alcoholic liver disease mortality data and population-at-risk were retrieved by county, gender, and age (10 year intervals). Aggregated, non-alcoholic liver disease mortality counts and population-at-risk were also retrieved by gender and age groups for the US to be used as the standard population in calculating non-alcoholic liver disease mortality rates, adjusting for effects of gender and age. Gender and age adjusted rates were calculated using indirect standardization for each county. Rate adjustment removes the effects of gender and age from crude rates in order to allow meaningful comparisons across populations with different underlying race and age structures. Population data from the US, during the study period, were used as standard populations to obtain a standardized rate for each county. Some other potential confounders, such as educational level and race, were also adjusted by putting the two factors as covariates in the regression model. The percentage of people over 25 with a college degree or above was used as an indicator of educational level and the percentage of black people was used to adjust for race. The percentage of people over 25 with a college degree was from the US Census Bureau, 2006–2010 American Community Survey and the percentage of black people was retrieved from the Multiple Cause of Death data in the CDC WONDER online database.

With the indirect standardization, the expected number of non-alcoholic liver disease deaths was first calculated for each county, which was determined by the number of cases that would be expected if people in the study population had the same mortality rate as people in the standard population with the same age and gender. Standardized mortality rates (SMRs) were calculated by dividing the observed count by the expected value. Death counts were “suppressed” when the data met the criteria for confidentiality constraints. Rates were suppressed for sub-national data representing zero to nine (0–9) deaths. Thereafter, all counties with suppressed data were not included for standardized rate calculation, spatial analysis, and modeling. The number of counties in the study area was 3109, with 195 counties being omitted that had suppressed disease data. Consequently, the number of data points (counties) for the statistical modeling was 2914. The suppressed data may lead to a bias of the model and the results needs to be interpreted with caution.

### Testing the hypothesis that non-alcoholic liver disease is related to bloom coverage

A flexible-shaped spatial scan statistic (FlexScan) was performed to identify spatial clusters of non-alcoholic liver disease deaths. Tango and Takahashi [45] showed that FlexScan detects irregular shaped clusters by using a limited exhaustive search that would detect arbitrarily-shaped clusters by aggregating their nearest circular neighboring areas.

Exploratory spatial data analysis and Bayesian regression models were used to assess the association between cyanobacterial blooms and the SMR of non-alcoholic liver disease using GeoDa software [46,47] and WinBUGS [48]. For exploratory spatial data analyses, a bivariate global Moran’s I statistic and local indicator of spatial association (LISA) were used. Bivariate global Moran’s I value determines the overall strength and direction of the relationship between the two variables, SMR and bloom coverage in each county. LISA provides information relating to the location of spatial clusters and outliers. Local statistics are important because the magnitude of spatial autocorrelation is not necessarily uniform over the study area. The LISA analysis by GeoDa presented a cluster map and identified clusters of High-High non-alcoholic liver disease clusters (units of significantly high disease mortality rates surrounded by significantly high bloom coverage), Low-Low clusters (units of significantly low disease mortality rates surrounded by significantly low bloom coverage), High-Low or Low-High outliers and insignificant areas (units where the relationship between disease mortality rates and bloom coverage were not significant). Significance was tested by comparison to a reference distribution obtained by random permutations; 999 permutations were used to determine a significance level for the differences between spatial units. Spatial contiguity was assessed as Queen’s contiguity that defines spatial neighbors as those areas with shared borders and vertices.

A negative binomial regression analysis was performed using STATA 13.0 (Stata Corp., College Station, TX, USA) to assess the relationship between non-alcoholic liver disease deaths and bloom coverage, adjusting for educational level and race. Negative binomial regression was used instead of Poisson regression because of the over-dispersed data. Thereafter, Bayesian negative binomial models were fitted in WinBUGS [48] to examine the association between non-alcoholic liver disease deaths and bloom coverage using a conditional autoregressive (CAR) process. Basically, spatial random effects were used at a county level to account for spatial correlation present in the data. Markov Chain Monte Carlo simulation (MCMC) was applied to estimate model parameters [49]. After the initial burn-in of 5,000 iterations, another 10,000 iterations were used for the summaries of the posterior distribution of the parameters. It was assumed that the observed counts of non-alcoholic liver disease deaths (*Yi*) in county *i* follow a negative binomial distribution with parameters *pi* and *r*; i.e., Y*i* ~ NB (p_i_, r), where p_i_ relates to the average number of cases via the formula (μ*i*) = *pit*/*r* where r is the over-dispersion parameter. We modeled the average number of deaths (μ*i*) as a function of potential risk factors as in the following:

$$ \mathrm{Log}\kern0.5em \left(\mu i\right)\kern0.5em =\kern0.5em  \log \kern0.5em Ei\kern0.5em +\kern0.5em a\kern0.5em +\kern0.5em  Xi\kern0.5em *\beta +ei+\kern0.5em \phi i $$

where *ui* denotes expected number of deaths in county *I;* α is the incidence rate when all covariates have zero value; ***X****i* is a vector of covariates in county *I*; β is a vector of the regression coefficients; e*i* is the unobserved (i.e., uncorrelated) heterogeneity; and *øi* is the structured spatial random effect. County-specific random effects were modeled via a conditional autoregressive (CAR) process, which implies that each *øi,* conditional on its neighbors, follows a normal distribution with a mean equal to the average of neighboring spatial effects, and variance is inversely proportional to the number of neighbors.

## Results

### Spatial distribution of cyanobacterial blooms in the contiguous US

**Figure 1:**
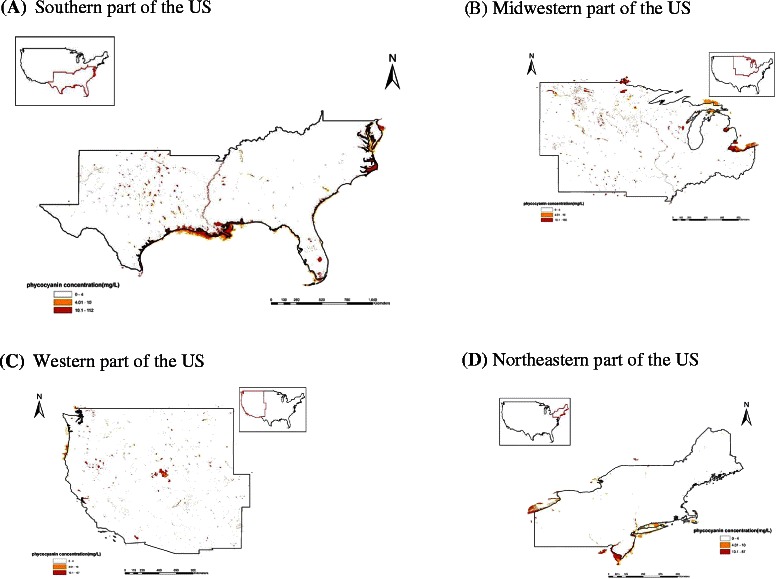
The spatial distribution of cyanobacteria blooms in different parts of the Contiguous US in 2005 as estimated by MERIS. **(A)** Southern part of the US **(B)** Midwestern part of the US **(C)** Western part of the US **(D)** Northeastern part of the US.

**Figure 2:**
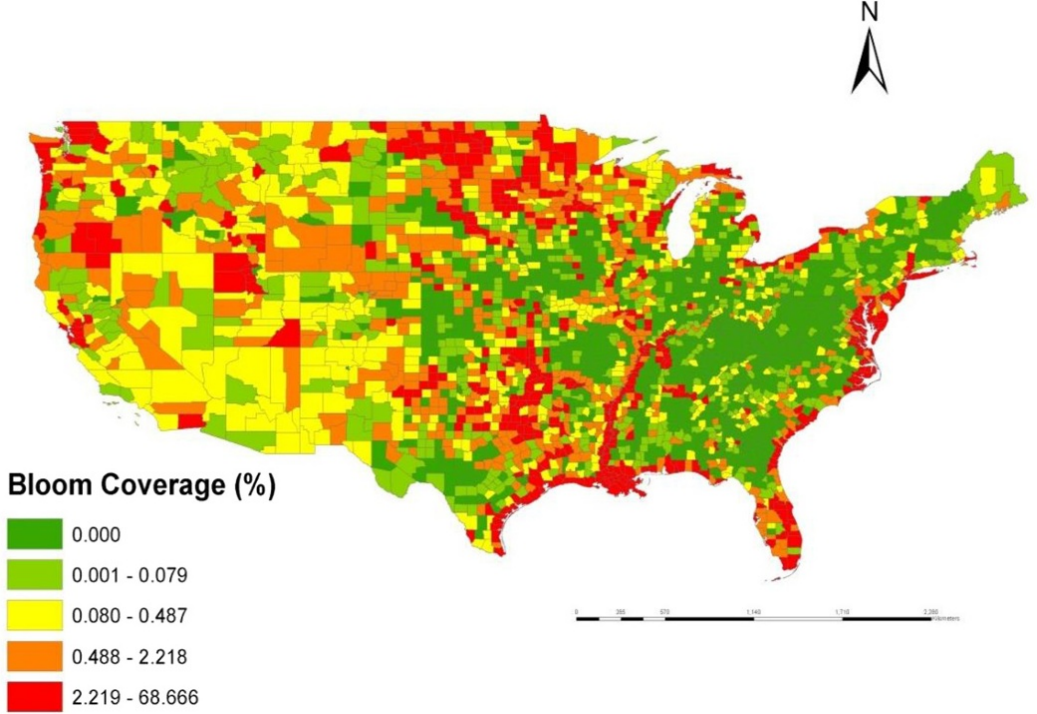
Bloom coverage area (percentage by county) in the US in 2005 as estimated by MERIS.

Based on the estimated phycocyanin concentrations from MERIS, it was observed that cyanobacterial blooms were widely spread in US water bodies, including lakes and rivers (Figure 1). From the maximum value composite image, it is evident that a large part of Lake Erie was covered by cyanobacterial bloom, mostly in the western basin. Other parts of the Great Lakes, such as Saginaw Bay, also showed significant bloom coverage. The largest lakes in the contiguous US (i.e. Great Salt Lake, Lake of the Woods, Lake Oahe, Lake Okeechobee, and Lake Pontchartrain) were all afflicted with cyanobacterial blooms. Coastal areas in Texas, Louisiana, North Carolina, Virginia, Maryland and Delaware also had significant cyanobacterial blooms. Based on our satellite estimations, the occurrence of cyanobacterial blooms in US waters was shown to be a common and serious problem. When the data were aggregated at the county level (Figure 2), it was observed that counties in coastal areas, as well as counties in the mid-north areas, have substantial bloom coverage; overall, 1,949 counties showed some bloom coverage, which is 62% of all the counties assessed. Due to the limitation of the spatial resolution of MERIS images, large lakes and estuaries were better represented than relatively small ponds. It is possible that the bloom situation was underestimated in areas where most water bodies were relatively small ponds.

### Spatial clusters of non-alcoholic liver disease

**Figure 3:**
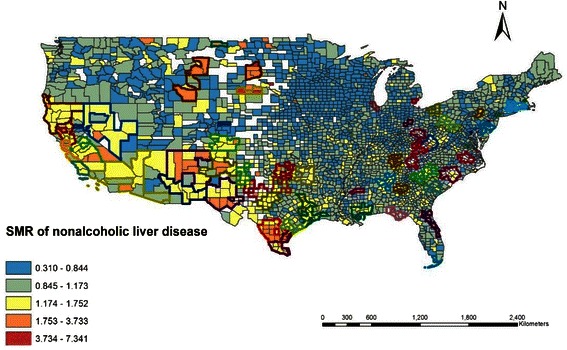
SMR of nonalcoholic liver disease of each county. The FlexScan identified significant clusters of death counts due to nonalcoholic liver disease from 1999 to 2010.

In total, 773,828 non-alcoholic liver disease deaths in the US from 1999 to 2010 were reported; a spatial variation in non-alcoholic liver disease mortality was observed (Figure 3). FlexScan identified 65 significant spatial clusters of non-alcoholic liver disease (*p* < 0.01), which included 432 counties. There were 26 significant clusters along the coastal areas versus 39 significant clusters in the inland areas. The most likely clusters were located along the coastal area of Texas and included 14 counties (*p* = 0.001). Counties in the clusters also showed higher bloom coverage than counties from the non-clusters according to the Wilcoxon signed-rank test (*p* < 0.001).

### Exploratory spatial analysis on the relationship between non-alcoholic liver disease and bloom coverage

**Figure 4:**
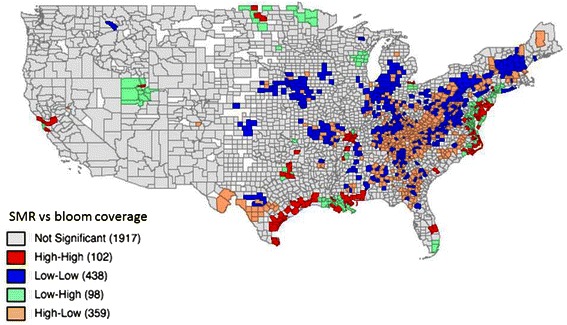
Bivariate LISA cluster map of nonalcoholic liver disease and cyanobacterial bloom coverage. The numbers of counties by the categories are shown in the parentheses.

The global Moran’s I value is 0.001 (*p* = 0.001), which indicates an overall positive spatial correlation of non-alcoholic liver disease SMR and bloom coverage. The bivariate LISA cluster map is shown in Figure 4 (permutations = 999, *p* <0.05), which shows local patterns of spatial correlation at the county level between SMR and average bloom coverage for its neighbors. Significant clusters, as well as outliers, are color coded by type of spatial autocorrelation. The High-High and Low-Low counties represent spatial clusters, while the High-Low and Low-High counties represent spatial outliers. The legend also shows the number of counties in each category. The clusters were observed in those places with significant positive spatial relationships between the two variables, while the outliers showed significant negative spatial relationships. High-High areas shown in Figure 4 tend to have high bloom coverage that is shown in Figure 2 as well as high SMR of nonalcoholic liver disease SMR shown in Figure 3. In contrast, High-Low areas shown in Figure 4 tend to have high bloom coverage shown in Figure 2, but low nonalcoholic liver disease SMR as shown in Figure 3. There were significant clusters around coastal areas near Texas.

### Bayesian regression of non-alcoholic liver disease on bloom coverage

Bayesian regression revealed a significant relationship between non-alcoholic liver diseases and bloom coverage using a negative binomial model (Table 1), accounting for the spatial correlated parttern of the data. According to this model, risk for non-alcoholic liver disease death increased 0.3% (95% Bayesian confidence interval, 0.1% to 0.5%) for each 1% increase in bloom coverage of the county; adjusting for age, gender, educational level, and race. In the US, if the bloom coverage per county increases by 1%, the estimated number of deaths per year will increase by about 440, given the current non-alcoholic liver disease death rate of 468 per 1,000,000 people per year. The results show that bloom coverage was a significant factor influencing the rate of non-alcoholic liver diseases.

**Table 1:** **Model estimates of the ajusted association between cyanobacterial bloom coverage and nonalcholic liver disease mortality in the Coutigious US by Baysian negative binomial regression**

Variable	Risk Ratio Estimate	95% credible interval
County bloom coverage	1.003	(1.001,1.005)
Percentage of adults with higher degree	0.385	(0.350, 0.422)
Percentage of black population percentage	1.60	(1.46,1.78)

## Discussion

We estimated the overall spatial distribution of cyanobacterial blooms in the contiguous US using MERIS-based phycocyanin levels. Harmful cyanobacterial blooms may be more common than previously estimated [50], as most large lakes in the US and coastal areas showed cyanobacterial blooms indicating a serious environmental problem in wide areas. In the contiguous US, 1,949 counties showed at least some blooms in their water bodies (due to the limitation of spatial resolution, small lakes could not be assessed). For monitoring of algal blooms and their toxins, it appears that remote sensing is a useful, quick, and cheap method for evaluation of large areas and can serve as a supplement to in situ monitoring of water bodies with more extensive coverage. Currently, a number of states regularly monitor cyanobacteria and cyanotoxins in water bodies (e.g. New York State implemented the “Citizens Statewide Lake Assessment Program” to monitor lake conditions, including harmful algal blooms and Ohio regularly monitors microcystin levels at recreational beaches) (http://epa.ohio.gov/ddagw/HAB.aspx). In the US, the distribution of mortality from non-alcoholic liver disease seems to vary geographically, which could be the result of possible environmental risk factors. In the contiguous US, we have identified 65 spatial clusters with high mortality rates for non-alcoholic liver disease; counties in the spatial clusters also showed higher cyanobacterial bloom coverage than counties in the non-clusters, indicating that environmental risk is associated with cyanobacterial bloom and could be contributing to the spatial clusters of non-alcoholic liver disease. By Bayesian spatial regression, we found a significant positive association between the relative risk of non-alcoholic liver disease mortality and cyanobacterial bloom coverage after adjusting for gender, age, race, and educational level; there was an excess risk of non-alcoholic liver disease mortality in those areas with high bloom coverage. The results show that spatial distribution of cyanobacterial blooms, estimated by remote sensing, was associated with non-alcoholic liver disease mortality, strongly suggesting that cyanobacterial blooms are an important risk factor.

Monitoring of phycocyanin data, obtained from remote sensing, can be used as an indicator of cyanobacterial blooms over a large area and can aid in assessing health risks due to theseblooms; cyanobacterial blooms may produce toxins, such as microcystins that have been shown to be liver toxins [23]. Living in bloom areas increases the probability that persons will be exposed to excess microcystin through inhalation, recreational exposure, or ingestion of contaminated food or water. Several cyanotoxins, such as microcystins, Nodularins and Cylindrospermopsin have shown to cause liver damage [51] and microcystins can cause liver hemorrhage and chronic effects, such as tumor promotion [51]. Microcystins have over 80 variants and could be produced by *Microcystis*, *Anabaena*, *Nostoc*, *Oscillatoria*, and *Hapalosphon* [9]. Nodularins are more commonly isolated from the filamentous, planktonic cyanobacterium, *Nodularia spumigena* are structurally similar to microcystins and can induce similar toxic effects [9,51]. Cylindrospermopsin causes liver hemorrhage [52] and can be produced by *Aphanizomenon*, *Cylindrospermopsin*, *Umezakia* [9]. Cyanobacterial blooms have also been implicated as a potential risk factor for amyotrophic lateral sclerosis [53].

This current study shows a possible association between non-alcoholic liver disease and spatial distribution of cyanobacterial blooms. Although such a study is generally suitable to show an association, it is not suitable to prove or disprove an etiological cause for disease. However, it can be used for hypothesis generation and testing. Other studies are needed to investigate the level of exposure through different routes that are sufficient to cause disease.

The significant association shown herein, between cyanobacterial blooms and non-alcoholic liver disease provides some evidence for a potential health risk, but more epidemiological research is warranted in order to more accurately assess this risk. Exposures and possible health effects (both acute and chronic) of cyanobacteria and their toxins need to be evaluated more extensively than under current conditions [54], especially now that global warming will be more favorable for cyanobacterial bloom-forming events. In developed countries, where people are collecting water from surface sources to drink, more actions should be taken to control bloom formation as microcystins are highly stable in water and resistant to boiling.

This study highlights several important points for consideration. First, large scale ecological studies, suchas that presented herein, are particularly useful under conditions where disease data at individual levels are not available and individual levels of exposure are difficult to obtain [55]. Second, the statistically significant positive association between non-alcoholic liver disease mortality rates and cyanobacterial coverages can be taken as a probable indication of a potential health effect. This association justifies the need for further studies to investigate the biological mechanism(s) responsible for the adverse effects of cyanobacterial toxins on human health, especially liver damage and liver disease. Third, the data show that satellites offer tremendous spatial coverage and provide a great resource for regional environmental monitoring, pollution event warnings, and environmental health studies. Satellite-estimated environmental factors could be used for studying potential health risks as demonstrated in this study (association between satellite bloom data and liver disease mortality). In the United States alone, toxic cyanobacterial blooms result in substantial losses of $2.4-4.6 billion annually in recreational, drinking, and agricultural water resources [56]. The economic costs of toxic cyanobacteiral blooms would be understandably more, if losses due to cyanobacterial blooms on health could be reliably estimated.

There are some limitations related to this study: 1) while effects were adjusted for gender, age, race, and educational level, other potential confounding factors were not included (e.g. obesity, smoking, Hepatitis B infection, and diet); 2) the study used aggregated data and therefore inferences based on the analysis cannot be directly transferred to an individual level, ecological studies, as presented here, i do not have the ability to distinctively incorporate individual information, as satellite measurements do not represent individual exposure due to differences in diet, recreational activities, etc.; 3) ENVISAT MERIS imageries have a spatial resolution of approximately 300 m, which limits the ability to assess small lakes and ponds, as well as data outages near coastal regions, which could lead to some biases in the estimation of exposure levels; 4) the spatial resolution limitation may lead to an underestimation of the bloom coverages in inland areas with more small ponds; 5) the population-based ecological study does not consider population dynamics during the study period (e.g. people may have migrated during the study period) and the residence at time of death may not be the location where the disease was initiated. According to the US 2000 census data, between March 1999 and March 2000, 43.4 million Americans moved and 39% of all moves were cross county [57]. We might underestimate the effect of bloom on nonalcoholic liver disease if impacted people moved away from the bloom areas; and 6) we used one year’s cyanobacterial bloom situation to represent a 12 year bloom situation. It is possible that the bloom situation could change quickly in a few years in some locations; however, it is rare. We could have potentially underestimated the bloom situation in areas where eutrophication increased very rapidly and miss some areas with both high bloom coverages and high non-alcoholic liver disease mortality. We may have underestimated the effect of bloom if the missing high bloom areas (remotely sensed) tend to have high nonalcoholic liver disease mortality rates. Alternatively, we may have overestimated the effect of bloom, if the missing high bloom areas (remotely sensed) tend to have low nonalcoholic liver disease mortality rates. Finally, the mismatch of the temporal window of remote sensing images and disease data could lead to potential bias in the results. In addition, coastal areas may suffer from bloom contamination resulting from adjacent waters, whose effects were not considered in this study and which may lead to an underestimation of the effects of bloom on non-alcoholic liver disease in coastal areas. Although most coastal areas close to blooms, also showed high bloom percentage, it is possible we may underestimate the bloom level for the coastal areas by using bloom coverage. Due to these study limitations, the association between cyanobacterial bloom and non-alcoholic liver disease should be interpreted with caution. It is obvious that more research is needed to confirm the effects of cyanobacterial blooms on liver disease as described herein.

## Conclusions

This ecological study in the contiguous US, using satellite data and data of multiple causes of death, found a significant positive association between risk of non-alcoholic liver disease mortality and cyanobacterial bloom coverage. We identified clusters of non-alcoholic liver disease mortality in clusters in those counties that also had higher bloom coverage.

The evidence for excess non-alcoholic liver disease in areas with high cyanobacterial bloom coverage suggests that more attention should be centered around the public health impact of harmful cyanobacterial blooms. Additionally, remote sensing could be used to efficiently monitor the distribution of algal blooms over a national or global level and serve as a possible early warning tool for public health alerts.

## Abbreviations

SMR: Standardized mortality rate
LISA: Local indicator of spatial association (LISA)
BMAA: Beta-methylamino alanine (BMAA)
NASA: National Aeronautics and Space Administration
GSFC: Goddard Space Flight Center
BEAM: Basic ERS & Envisat (A) ATSR and MERIS (BEAM)
TOA: Top of atmosphere
R_LW: Water leaving radiance
WHO: World Health Organization
CDC: Centers for Disease Control and Prevention
WONDER: Wide-ranging Online Data for Epidemiologic Research
FlexScan: flexible-shaped spatial scan statistic
CAR: Conditional autoregressive
MCMC: Markov Chain Monte Carlo simulation
MERIS: The Medium Resolution Imaging Spectrometer
ENVISAT: Environmental Satellite
ESA: The European Space Agency
